# Left ventricular ejection fraction by multigated acquisition scan using planar sodium iodide and cadmium-zinc-telluride cameras: a comparison with two-dimensional echocardiography

**DOI:** 10.22038/AOJNMB.2022.60392.1424

**Published:** 2023

**Authors:** Gouri Kumar Das, Chen Siew NG, Mahayuddin Abdul Manap

**Affiliations:** 1 Department of Nuclear Medicine, Hospital Sultanah Aminah, Johor Bahru, Johor, Malaysia; 2 Oncological and Radiological Sciences Cluster, Advanced Medical and Dental Institute, Universiti Sains Malaysia, Bertam, Penang, Malaysia

**Keywords:** Breast cancer, LVEF, 2D-echocardiography, MUGA, Planar NaI camera, CZT camera

## Abstract

**Objective(s):**

This study was undertaken to compare the correlation and agreement between Modified Simpson’s method two-dimensional-echocardio-graphy (2D-echo) and rest multigated acquisition scan (MUGA) using both planar sodium iodide (pNaI) and cadmium-zinc-telluride (CZT) cameras to measure left ventricular ejection fraction (LVEF).

**Methods:**

One hundred and nine breast cancer patients monitored for cardiotoxicity underwent 2D-echo, followed by pNaI and CZT MUGA scans on the same day. LVEF for CZT camera was processed using both automatic and manual processing methods, thus yielding four methods for the LVEF analysis.

**Results:**

Significant correlation (p<0.01) was seen among all four methods, with varied correlation strengths. Moderate correlation was seen between 2D-echo and both pNaI (r=0.56) and CZT cameras (automatic r=0.54, manual r=0.56). Strong correlation was registered between pNaI and CZT camera (automatic r=0.72, manual r=0.71). Bland-Altman limits of agreement among the three scans were wide and suboptimal. The widest limits were -21.1 to +16.2 (37%) between 2D-echo and CZT auto-processing.

**Conclusion:**

Any one of the modalities can be used to measure LVEF, however, their results should not be used interchangeably. The same method of measurement is advised for serial scans.

## Introduction

 Cardiotoxicity has emerged as a major concern in cancer patients undergoing chemotherapy or targeted therapy (1, 2). This is especially the case for breast cancer patients for whom early detection and treatment have significantly improved survival rates. The most common manifestation of cardiotoxicity is the subtle reduction in left ventricular ejection fraction (LVEF), attended by a late manifestation of left ventricular dysfunction. Doxorubicin and Trastuzumab are the two most common drugs associated with cardiotoxicity. The majority of clinical practice guidelines (3-5) have recommended baseline as well as serial measurements of LVEF during treatment and to repeat scans 1-2 years post-completion of cardiotoxic treatment. A drop in LVEF by greater than 10% to a level below 50%, is consistent with early cardiotoxicity, and the chemotherapeutic drug is usually discontinued (6).

 Currently, the three most common methods used to measure LVEF are Modified Simpson’s method 2D-echocardiography (2D-echo), cardiac magnetic resonance imaging (CMR), and multigated acquisition scan (MUGA). Various studies (7-11) have regarded serial MUGA as the best modality for measuring LVEF, although currently, the gold standard is CMR (9, 12-15) due to its high spatial resolution, free of geometric assumptions, having no ionizing radiation, and useful in obese patients. However, CMR usually requires long scanning time, and being a high-cost modality, it is often unavailable in many smaller hospitals, thereby yielding the ground to cheaper options. Although these methods of measuring LVEF can show a significant linear relationship when compared, they need not necessarily be in agreement as noted by Bland and Altman (16, 17).

 An agreement between two methods evaluates the bias between the mean differences and provides an estimated agreement interval within which 95% of the differences between the two methods would fall. The narrower the agreement interval, the more interchangeable the techniques are to measure LVEF. To the best of our knowledge, this is the first study that compares on a pairwise basis the correlation and agreement among the Modified Simpson’s 2D-echo and the MUGA scans involving both planar and CZT (manual and auto-processing modes) cameras. We have also analyzed the interobserver variation between two nuclear medicine physicians measuring LVEF using the two different gamma cameras and measured the RVEF values acquired with the CZT camera among breast cancer patients.

## Methods

 This is a prospective, observational and comparative study.

***Patient recruitment and selection***

 A total of 109 female breast cancer patients were referred from the Oncology and Radiotherapy Department of Hospital Sultan Ismail, Johor Bahru for LVEF measurement as part of the assessment process in monitoring drug-induced cardiotoxicity. They comprised of patients who are receiving (n=84) or scheduled to receive (n=25) cardiotoxic treatment and were recruited between March 2018 – March 2019. Follow-up studies of the same patients were not included in the study. They first underwent 2D-echocardiography, followed by MUGA scans using both planar NaI and CZT cameras on the same day at the Cardiology and Nuclear Medicine Department of Hospital Sultanah Aminah, Johor Bahru respectively.

 Patients with BMI >35; irregular heart rhythm; left arm lymphedema; pregnancy; and history of underlying heart problems were excluded from the study. The patient characteristics of the study population are shown in Table 1. A total of 62 patients had left breast cancer and 47 patients had right breast cancer. 53 patients had undergone left breast surgery (either mastectomy or wide local excision) before the study. A total of 42 patients had received left chest wall radiotherapy before the scan, and 79 patients were on Trastuzumab therapy as well. 

 The average number of cycles of cardiotoxic treatment these patients received was 10 (range 1-32 cycles). The mean duration of time between receiving the cancer therapy and performing the scan was 19.4±3.7 days (range 11-30 days). This study was approved by the Medical Research & Ethics Committee, Ministry of Health Malaysia. Informed written consent was obtained from all the patients before the start of the study.

**Table 1:** Characteristics of the study population (N=109)

**Patient characteristics **	**Number of patients**	** Percentage**
**Mean age, years (range)**	53.0 ± 10.9	23 - 76
**Mean body mass index (range) **	25.5 ± 4.3	14.8 - 34.9
**Ethnicity **		
Malay	52	47.7
Chinese	49	45.0
Indian	8	7.3
**Gender**		
Female	109	100.0
**Type of surgery **		
Left Mastectomy	50	45.9
Left Wide local excision	3	2.8
Right Mastectomy	31	28.4
Right Wide Local Excision	16	14.7
No previous breast surgery	9	8.2
**Type of cardiotoxic treatment received **		
Herceptin (Trastuzumab)	75	89.2
Herceptin & Perjeta (Pertuzumab)	6	7.1
Anthracyclines	3	3.6
**Radiotherapy **		
Left chest wall radiotherapy	42	38.6
Right chest wall radiotherapy	31	28.4
No radiotherapy	36	33
**Type of scan **		
Baseline	25	22.9
Interim	84	77.1

***Scan methodology***

**Figure 1:**
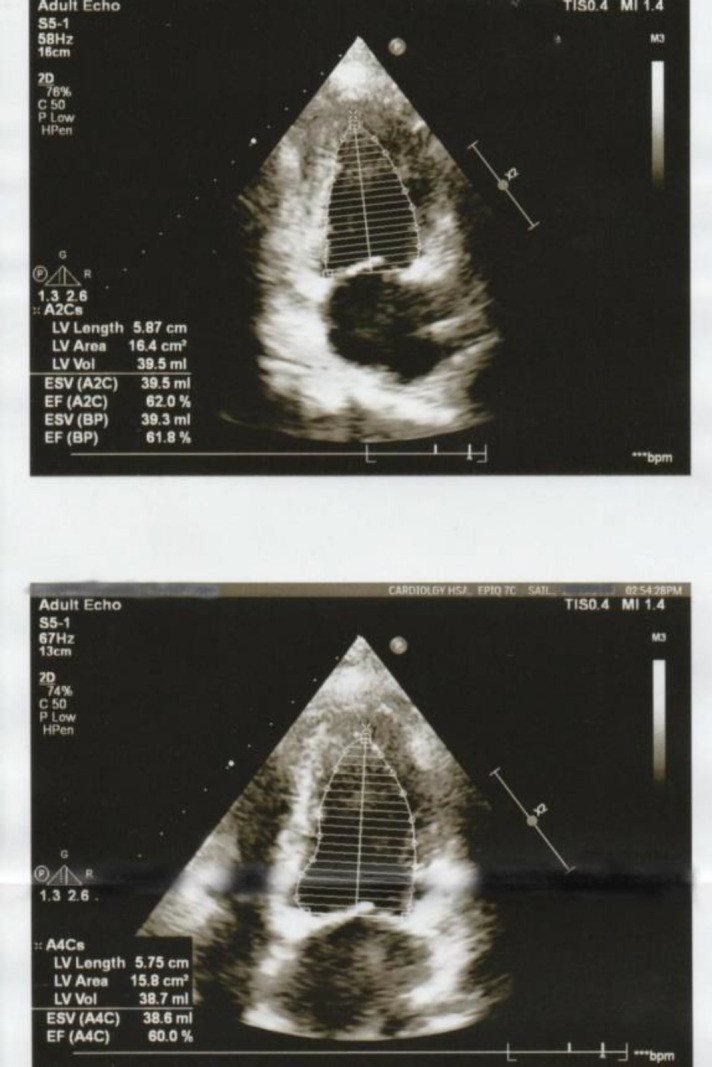
Modified Simpson's biplane method for measurement of ejection fraction in the apical two-chamber and four-chamber view

 For echocardiography, the Modified Simpson’s biplane method, in both two-chamber and four-chamber apical views (Figure1), was performed using the Philips Epic Series 7C machine. Using the disc-summation algorithm, the end-diastolic and end-systolic volumes were calculated to derive the ejection fraction.

The MUGA scans, which involved labelling the cardiac blood pool with Technetium-99m (^99m^Tc) radiotracer, were done on the same day following the 2D-echo study. The scans were performed using both the traditional 2D planar NaI camera and the CZT camera with higher spatial and contrast resolution (18, 19). The image acquisition with these cameras was ‘gated’ with electrocardiogram (ECG) to obtain information over several cardiac cycles.

**Figure 2:**
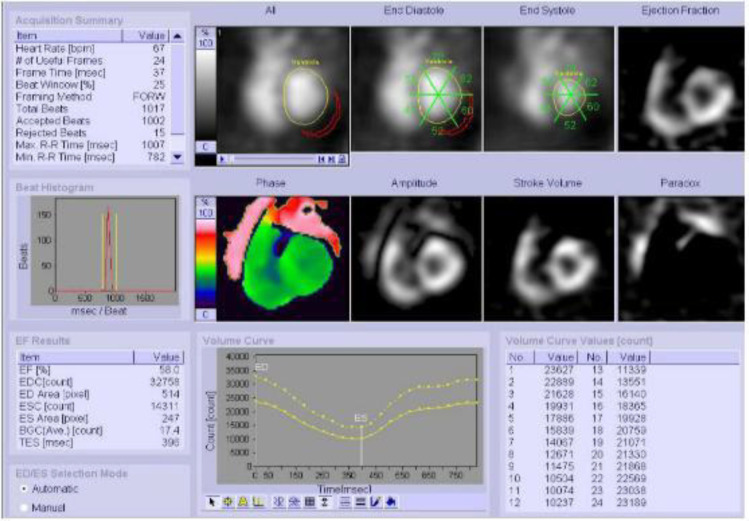
Semiautomatic region of interest (ROI) determination around the left ventricle and results of LVEF measurements by planar-NaI camera depicting both global and regional LVEF values

 The planar-NaI camera used was the Siemens Symbia E Dual Head camera (Siemens Medical Solutions Inc. USA). The processing method was semiautomatic, using the Siemens Gated Bloodpool Software and re-orientated with the Syngo MI application software. LVEF was calculated by drawing a region of interest (ROI) that was done by the software over the LV at end-systole and end-diastole, and the background at 5 o’clock position in the immediate proximity of LV (Figure 2). The ROI was checked for each frame and manual corrections were made when indicated. LVEF was calculated using the (20):

$\frac{\mathrm{Background corrected end}-\mathrm{diastolic counts}-\mathrm{Background corrected end}-\mathrm{systolic counts})}{\begin{array}{c} \left(\mathrm{Background corrected end}-\mathrm{diastolic counts}\right) \end{array}}$ ×100

**Figure 3:**
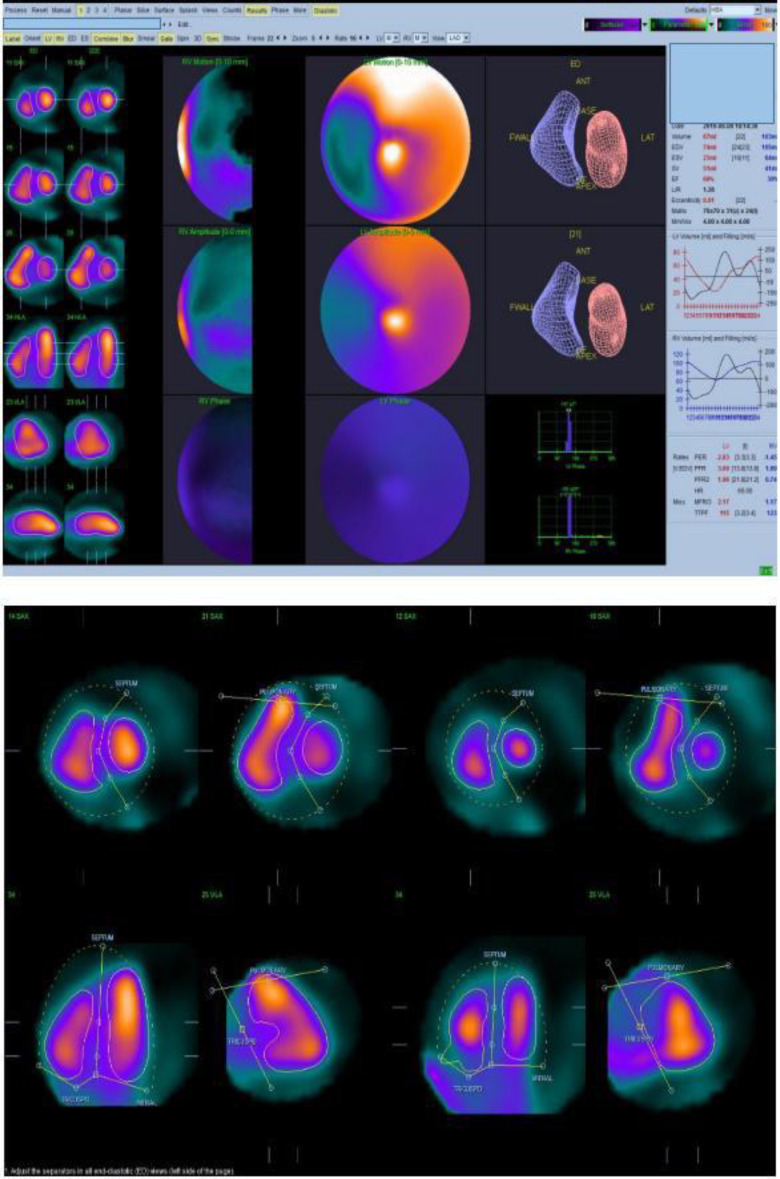
LVEF and RVEF measured by the 3D-CZT camera using QBS software with automatic processing(Top image) and manual processing (Bottom image)

 The CZT camera used was the Discovery NM 53°C (GE Healthcare, Milwaukee W1, USA), which has a multi-pinhole collimator system with an array of nineteen CZT pixelated detectors. The software used to process the LVEF was the Quantitative Blood Pool SPECT (QBS-Cedars Sinai, Los Angeles, C, version 2009) using both manual and automatic processing methods (Figure 3). The acquisition parameters for the two gamma cameras are given in Table 2.

**Table 2.** Acquisition Parameters of Planar and CZT cameras

	**Planar- NaI**	**SPECT CZT**
Acquisition mode	2D	3D
Dose of Tc-99m labelled RBC	20 mCi injected once - 30 minutes prior to the scan	
Collimator	LEHR (low energy high resolution)	Pinhole
Frames/bin	24	24
R-R interval	15-20%	15%
Acquisition time (min)	15	15
Requested accepted beats	900	600
Processing software	Siemens - Planar Gated Bloodpool Software	Cedars Sinar - Quantitative Blood Pool SPECT ( QBS)
Reorientation software	Syngo MI (Siemens)	Xeleris (GE)
Camera positioning	Single head 45º LAO	19 fixed detectors
Matrix size	64×64	74×74 resized to 64×64
Reconstruction algorithm	None	Generic
Reconstruction filtering	None	Generic
Intrinsic spatial resolution	3.48 mm	2.5 mm
System resolution	8.5 mm	<6.5 mm

 LVEF values for the planar NaI camera and the CZT camera (both manual and automatic processing methods) were analyzed by 2 doctors who were blinded to scan results of each other and also to the 2D-echocardiography results to assess the interobserver variability. The end-diastolic volume (EDV) and end-systolic volume (ESV) parameters were not used for comparative study as the method of computing LVEF using the planar NaI camera was based on activity counts rather than volume measurements.

***RBC Radiolabelling Methodology***

 Radiolabelling the patient’s red blood cells (RBCs) with ^99m^Tc-pertechnetate was done once using the in-vivo method. The dose of ^99m^Tc labelled RBC was 20mCi (740 MBq). Radiolabelling efficacy was checked 30 minutes later, and just before the start of radionuclide scans, by withdrawing 1-2 mL of blood from the patient. The radiolabelling efficiency of ^99m^Tc labelled RBC was 96.1±1.8% (range of 90-99%) among the study subjects. Although the in-vivo method of radiolabelling is known to have the lowest labelling efficiency compared to the modified in- vivo, Brookhaven in-vitro and the Ultratag method, the Society of Nuclear Medicine Procedure Guideline for Gated Equilibrium Radionuclide Ventriculography, 2002 (21) and the British Nuclear Medicine Society Procedure Guideline for Planar Radionuclide Cardiac Ventriculogram for the Assessment of Left Ventricular Systolic Function, Version 2, 2016 (22) have both stated that the in-vivo method is still acceptable for MUGA studies as it is a quick, simple and inexpensive method. The average reported labelling efficiency using the in-vivo method varies widely from 71-96% (23). The higher values can be related to different methods of defining “labelling efficiency”. The definition used in our study is based on the US Pharmacopeia Expert Committee, USP 29 guidelines (24), whereby blood samples are centrifuged and the fractions of blood pool activity in plasma and in red blood cells are calculated. This definition, however, does not take into account the ^99m^Tc that may have diffused into the extravascular space or localized in organs such as thyroid or stomach. The lower values found in other studies could therefore be due to the calculation of non-blood pool activity by calculating labelling efficiency as the fraction of injected ^99m^Tc activity bound to red blood cells(23). 

***Statistical analysis***

 Data analysis was performed using SPSS software version 24.0. One-way ANNOVA with post hoc Tukey’s test was used to determine the difference in mean LVEF between the 2D- Echo, planar-NaI, and the CZT (manual and auto-modes) camera. Pearson correlation coefficient was used to assess the correlation between the different modalities to measure LVEF as per Schober et al. (25): negligible (r< 0.10), weak (r=010-0.39); moderate (r=0.40-0.69); strong (r=0.70-0.89); very strong (r=0.90-1.00).

 Bland-Altman analysis (17) was used to determine the agreement between the LVEF values derived from 2D-Echo and MUGA scans by plotting the difference in LVEF values against the mean of the LVEF values between two compared modalities. The plot shows the limits and range of agreement (encompassing 2 standard deviations above and below the mean difference value). Linear regression analysis with t-test was done to look for proportional bias between the LVEF results of two compared methods; values of p>0.05 are indicative of the absence of any statistically significant proportional bias between results.

 To measure interobserver variability, the Interclass Correlation Coefficient (ICC) based on 95% confidence level was used and interpreted as per guidelines of Koo et al. (26): poor reliability <0.5; moderate reliability >>0.5-<0.75; good reliability >>0.75-<0.9; excellent reliability >>0.9.

 To study the effect of left breast attenuation on LVEF values, an Independent t-test was done to measure the difference in mean LVEF values among patients with and without the left breast tissue.

## Results

 In the real-world setting, a study of this nature involving breast cancer patients who receive or scheduled to receive cardiotoxic treatment, the patients have to be deemed fit in the first place by the oncologist to undergo cardiotoxic treatment. Thus the cohort of 109 patients sent over for the one year study, was taken in its entirety, and were, therefore, not subjected to a selection based on the preliminary findings of equal amounts of patients with normal and abnormal LVEFs. As it turned out in the study (see Table 3), the majority of the patients inadvertently showed a normal range of LVEF, with just but a few exceptions. Similar normal LVEF ranges were seen among patients ongoing cardiotoxic treatment (n=84), which is shown in Table 4.

**Table 3:** Study population with normal and abnormal LVEF values as categorized by the American Society of Echocardiography and the European Association of Cardiovascular Imaging

	**Normal LVEF**	**Mildly abnormal LVEF**	**Moderately abnormal LVEF**	**Severely abnormal LVEF**	**Overestimated LVEF**
	**54-74%**	**41-53%**	**30-40%**	**<30%**	**>>75%**
2D-echo	101 (92.7%)	5 (4.6%)	0 (0%)	1 (0.9%)	2 (1.8%)
CZT-auto	81 (74.3%)	11 (10.1%)	1 (0.9%)	1 (0.9%)	15 (13.8%)
CZT- manual	72 (66.1%)	3 (2.7%)	0 (0%)	1 (0.9%)	33 (30.3%)
p- NaI camera	76 (69.8%)	25 (22.9%)	1 (0.9%)	1 (0.9%)	6 (5.5%)

**Table 4:** Mean LVEF values among patients that received cardiotoxic cancer therapy

	**Mean LVEF (%)**	**Standard deviation (%)**	**Range (%)**
2D-Echo	61.4	4.8	45-70
CZT -auto	63.4	9.9	38-90
CZT- manual	69.6	8.7	47-95
p- NaI camera	58.5	8.9	38-91

n=84

**Figure 4:**
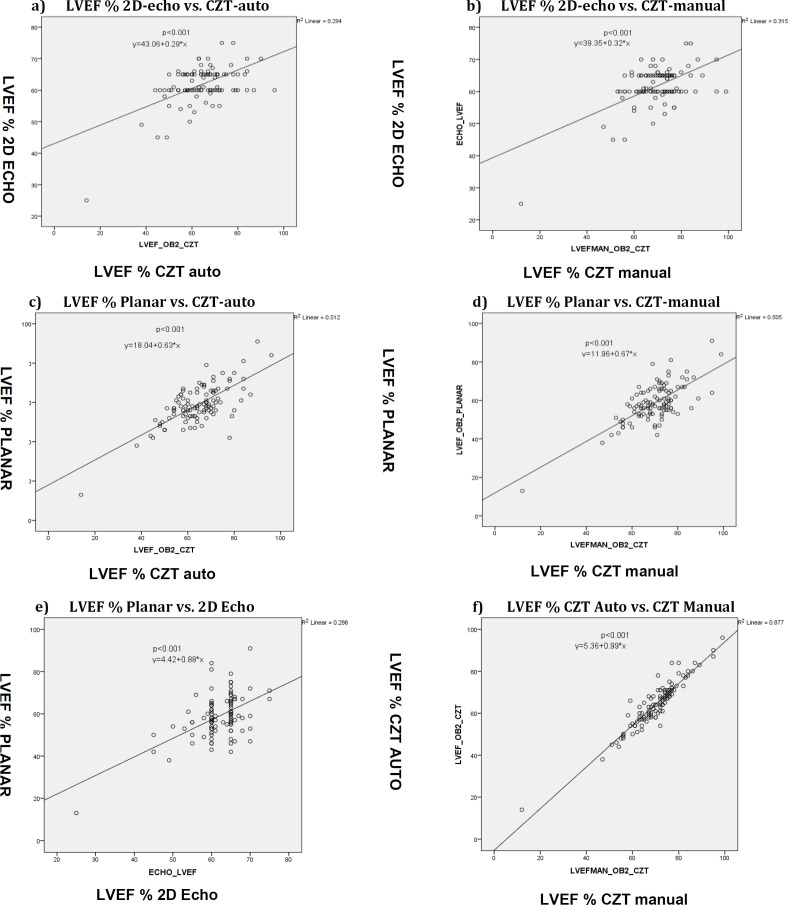
(**a- f**). Linear correlation plots of LVEF with (**a**) 2D-echo vs CZT-auto, (**b**) 2D-echo vs CZT- manual, (**c**) planar-NaI vs CZT- auto, (**d**) planar-NaI vs CZT- manual, (**e**) planar-NaI vs 2D-echo, (**f**) CZT- auto vs CZT- manual

 The highest mean LVEF measured was for CZT-manual with an LVEF of 70.1±10.7% (range 12-99%) and the lowest mean LVEF was 58.8±10.0% (range 13-91%) for planar NaI camera. The mean LVEF was 64.3±11.3% (range 14-96%) for CZT-auto and 61.8±6.1% (range 25-75%) for 2D-echo. Table 5 compares the differences in the mean values among paired modalities. The mean LVEF values were significantly higher for CZT-manual and auto-processing compared to planar NaI camera (p<0.001). The mean difference of LVEF values generated by these 4 different modalities of investigation showed no statistically significant difference when 2D-echo was compared to CZT-auto (p=0.24) or planar NaI camera (p=0.101). Figure 4 (a-f) shows the graphical representation of the Pearson correlation between the modalities. There was a significant correlation (p<0.01) among all four methods, but the correlation strengths were varied. Moderate correlation was seen between Modified Simpson’s 2D-echocardiogram and the other modalities: pNaI camera (r=0.56); CZT automatic processing (r=0.54) and CZT manual processing (r=0.56).

**Table 5:** Pairwise comparison of modalities in LVEF determination with One way ANNOVA

**Group 1**	**Group 2**	**Mean Difference** **(Group 1 -Group 2)**	**Standard Error**	**Significance** p value *	**95% Confidence Interval**	
					**Lower Limit**	**Upper Limit**
2D-Echo	CZT-Auto	-2.47	1.32	0.242	-5.87	0.94
	CZT- Manual	-8.25	1.32	<0.001	-11.67	-4.85
	p-NaI	3.03	1.32	0.101	-0.38	6.43
CZT- Auto	2D-echo	2.47	1.32	0.242	-0.94	5.87
	CZT- Manual	-5.78	1.32	<0.001	-9.18	-2.38
	p-NaI	5.49	1.32	<0.001	2.09	8.90
CZT- Manual	2D-echo	8.25	1.32	<0.001	4.86	11.65
	CZT- Auto	5.78	1.32	<0.001	2.38	9.18
	p-NaI	11.28	1.32	<0.001	7.88	14.7

* Mean difference is significant at the 0.05 level

**Figure5 (a-f):**
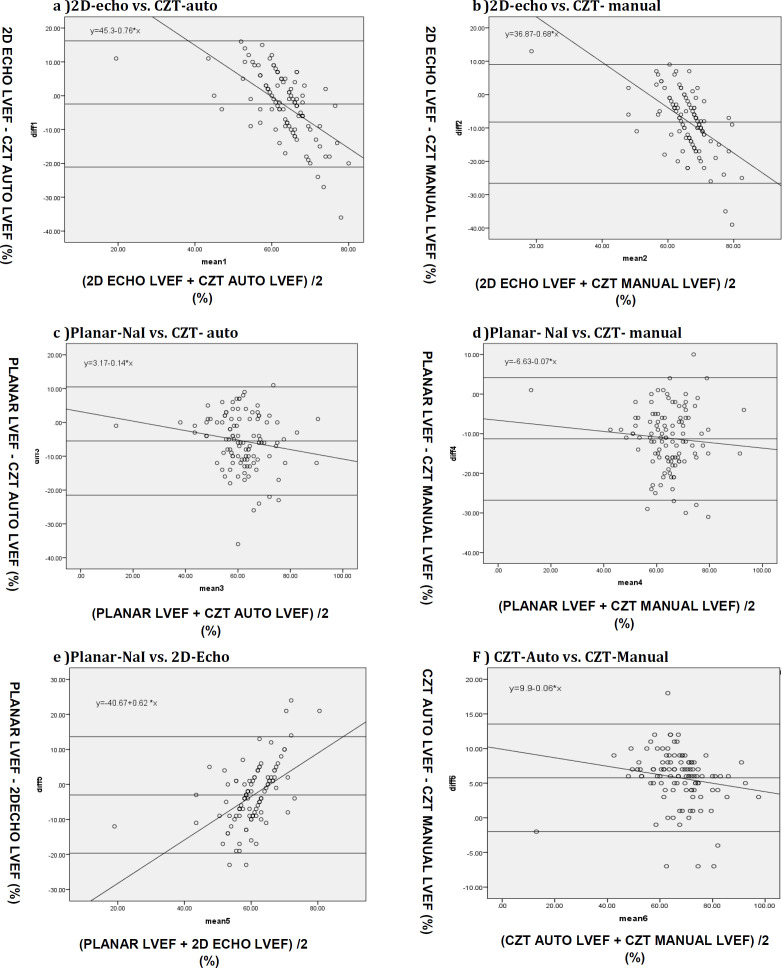
Bland-Altman plots with linear regression analysis of the agreement between cameras regarding LVEF for (**a**) 2D-echo vs CZT-auto, (**b**) 2D-echo vs CZT- manual, (**c**) planar-NaI vs CZT- auto, (**d**) planar-NaI vs CZT- manual, (**e**) planar-NaI vs 2D-echo, (**f**) CZT- auto vs CZT- manual

 A strong correlation was observed between pNaI and CZT cameras, with r=0.71 for manual processing and r=0.72 for auto-processing. The strongest correlation was manifested between the CZT automatic processing and manual-processing methods (r=0.94). The Bland-Altman plots with linear regression analysis aimed at examining the level of agreement among the modalities are depicted in Figure 5 (a-f), and the data are collected in Table 6. The Bland-Altman range or limits of agreement were found to be wide and suboptimal between all the scans. The interobserver variability between two physicians measuring the LVEF values using planar NaI and the CZT camera (both manual and automatic processing) and the mean LVEF values for each modality independently calculated by them are presented in Table 7. The mean RVEF value among breast cancer patients in this study assessed by CZT-auto was 41.5±8.6%, with a normal reported range of 19-63%. With CZT-manual, the mean RVEF was 40.2±8.9%, with a normal reported range of 17-65%. 

**Table 6:** Bland-Altman limits, total range of agreement (equal to ± 2 SD), and t- test linear regression analysis for proportional bias, from plotted LVEF data in pairwise comparisons of modalities

**Method**	**Bland-Altman Limits %**	**Bland- Altman Range %**	**Linear Regression Analysis** ** p value**
2D-echo vs CZT-auto	-21.1 to +16.2	37	<0.001
2D-echo vs CZT-manual	-25.6 to +9.06	35	<0.001
p-NaI vs CZT- auto	-21.5 to +10.5	32	0.08
p-NaI vs CZT-manual	-26.8 to +4.2	31	0.37
p-NaI vs 2D-echo	-19.7 to +13.6	33	<0.001
CZT-auto vs CZT-manual	-2.0 to +13.5	15	0.08

**Table 7:** Interobserver variation in MUGA scans analysed by two different Nuclear Medicine Physicians

**Method**	**Mean LVEF**	**Average measures of Interclass correlation (26)**	**95% confidence interval**
**CZT- Auto**		0.995	0.992-0.997
Observer 1	64.7±11.3%		
Observer 2	64.3±11.3%		
**CZT- Manual**		0.975	0.964-0.983
Observer 1	70.0±10.4%		
Observer 2	70.1±10.7%		
**p-NaI camera**		0.991	0.985-0.994
Observer 1	58.2±10.4%		
Observer 2	58.8±10.0%		

 A finding in our study involving measurements by CZT camera (auto and manual processing), showed that 18 patients (16.5%) had small LV cavities (ESV<20 mL). A total of 8 out of the 18 patients assessed using CZT-auto showed LVEF>80%, and the highest recorded was 96%. A similar high overestimation of the LVEF and higher LVEF values (up to 99%) was seen with CZT-manual. Overestimation of LVEF was also seen using the planar camera; the highest LVEF value recorded was 91%.

 The mean LVEF values for 42 patients that had left chest wall or breast radiotherapy before enrolment in the study, were 62.3±4.2% (range 54-75%) when measured with 2D-echocardiography; 58.0±8.2% (range 43-81%) for the planar NaI camera method; 65.0±10.0% (range 44-87%) and 70.6±8.9% (range 54-95%) respectively for CZT camera with automatic and manual processing methods. The mean LVEF values among these patients were comparable to the 36 patients that did not receive radiotherapy in this study.

 Of the 109 breast cancer patients in this study, 50 patients had undergone left mastectomy surgery, while the remainder had preserved left breast tissue (including those that had left wide local excision breast surgery). The types of surgery that patients underwent before enrolling in the study are shown in Table 1. The mean LVEF values and difference of mean LVEF values among this group of patients are summarized in Table 8.

**Table 8:** Mean LVEF values and difference in mean LVEF values (Independent t-test) among patients with and without left breast tissue

	**Left breast mastectomy** **n=50**	**Left breast tissue preserved** **n=59**	Difference in mean p value *
2D-Echo (mean LVEF, range)	62.5±3.9% (54-75%)	61.2±7.4% (54-75%)	0.09
CZT –auto (mean LVEF, range)	64.1±9.9% (44-90%)	64.5±12.5% (14-96%)	0.57
CZT- manual (mean LVEF, range)	69.7±8.4% (53-95%)	70.3±12.3% (12-99%)	0.42
p- NaI (mean LVEF, range)	59.9±8.9% (43-91%)	58.3±10.7% (13-84%)	0.42

* Mean difference is significant at the 0.05 level

## Discussion

 When cardiac function is assessed on a serial basis to assess the cardiotoxic effects of cancer therapy, a more precise measurement is required as it is important to detect the change in LVEF confidently and accurately. The clinician needs to know if the change in LVEF is valid and does not occur solely by chance. With the various methods available to assess cardiotoxicity, the clinician needs to know the correlation and agreement between the various methods and, most importantly, if these methods are interchangeable and have low interobserver variability.

 The 2D-echo extrapolates data from a limited sampling of the left ventricle. The procedure is time-consuming, operator dependent and subjected to acquiring a good definition of endocardial border during the procedure (9). Inherent in the Modified Simpson’s method is the geometric assumption that the LV function displayed in 4-chamber and 2-chamber apical views accurately reflects the actual global LV function. However, in patients with extensive wall motion abnormalities, the LVEF can be overestimated by as much as 5.2% (p<0.001) and is often attended by an interobserver variability of 11% (27, 28).

 The MUGA scan with no geometric assumptions is the method of choice to detect regional and global LVEF. It has been used in recent times for diastolic measurements of LV function such as peak filling rate and dyssynchrony (29). Also, with the advent of the higher resolution CZT camera, a 3D calculation of different parameters, including LVEF, RVEF, LV volumes, and LV synchronicity, can be obtained. The important advantages of the CZT camera are that it allows for a dose reduction of the radiopharmaceutical used during MUGA scans, and it provides a faster acquisition time while maintaining diagnostic accuracy. The injected activity could be decreased by 60-80% (30). The study by Duvall et al. (30) using the similar CZT camera, reduced the imaging time to five minutes, and the injected activity was reduced to 4-8 mCi (1.0-2.1 mSv) compared to an average dose of 20 mCi with a 15-minute acquisition used in this study. This would be beneficial for breast cancer patients who would normally undergo 17 cycles of three weekly Trastuzumab in a year and would therefore require at least six MUGA scans during their treatment.

***Mean LVEF and differences among them***

 In our study, the generally higher LVEF values obtained with the CZT camera (both manual and automatic processing) are consistent with the findings of Mitra et al. (29). The lower LVEF values obtained with the planar-NaI camera also corroborate with the findings of Chen et al. (31) who noted a mean LVEF value of 55.7±22.7% which was attributed to lower count sensitivity and spatial resolution of the camera and the use of different acquisition parameters.

 Higher LVEF values were obtained with CZT-manual when compared with CZT-auto, p-NaI, and 2D-echo (p<0.001). The reason for higher LVEF with CZT- manual over CZT-auto could be due to the use of different algorithms within the same software and the subjective perception on the edge of the image contour when manually drawing the region of interest (ROI) over the ventricle.

 LVEF values were higher for CZT-auto than for p-NaI (p<0.001). It can be speculated that this difference may be due to variations in the acquisition setup, reorientation software, and image filtering (20). This finding was also noted in other studies (29, 30, 32) and is explicable in terms of the complete exclusion of left atrium intrusion into the frames of the LV for global ROI measurements with the CZT camera. This would seemingly give an edge to CZT over the planar camera for LVEF measurements. However, when transitioning from planar to CZT camera, one should caution against the continued use of planar LVEF normal reference values since these would be significantly lower than that expected for the new CZT cameras. This could give rise to false-positive results if both these scans were used interchangeably for serial assessment of LVEF. It is prudent that validation of both these scans is undertaken in our local institutions.

 There was no statistically significant difference between the mean LVEF values of 2D-echo with CZT-auto and with planar-NaI (p>0.05) in our study. In a contrasting study by Bellenger et al. (9), there was a significant difference between LVEF values of 2D-echo and planar-NaI (p<0.001) in heart failure patients. However, this could be due to limitations of the 2D-echo that assumes the heart is ellipsoid and not spherical as often seen in heart failure patients.

***Correlation and agreement of LVEF values***

 A very strong correlation with r=0.94 (p<0.001), a narrow Bland -Altman range of 15% implying good agreement, and the lack of any significant proportional bias as confirmed by linear regression analysis (p>0.05) was seen between CZT-auto and CZT-manual, despite CZT-manual showing significantly higher LVEF values than CZT-auto. This would imply that the automatic processing method would suffice to measure the LVEF for the CZT camera without the need to use the slightly more time-consuming manual processing method.

 There was strong correlation between planar-NaI and CZT-auto (r=0.72) and CZT-manual (r=0.71). However, the limits of agreement between p-NaI and CZT cameras were wide (31-32%) and argue against their interchange-ability. The wide limits would also mask any significant drop in LVEF and place the patient at risk if the treatment were to be continued. This has formed the basis for setting our institutional criteria of 15% as the cut-off value for interchangeability of any two modality results.

 A close examination of the relevant Bland- Altman plots (Figure 5c and d) show the LVEF values from p-NaI and CZT appear to be in agreement in the LVEF range of 40 to 80%, with linear regression analysis attesting to the absence of any statistically significant (p>0.05) proportional bias between the results. It would thus appear that the results from these two modalities are interchangeable, although they fall short of our 15% institutional cut-off criteria for acceptance. This suggests that either the planar-NaI or CZT methods can be used independently and adequately for gross determination of LVEF and to distinguish between severely or moderately impaired and normal LVEF values. However, their results are not interchangeable.

 The same Bland-Altman plots also showed that p-NaI and CZT are not in agreement at lower LVEF values <40%. Although, this study could not accommodate a higher number of patients with LVEF<40% to draw firm conclusions, nevertheless, this result arguably indicates that the planar camera overestimates, while CZT camera underestimates the LVEF values at this lower LVEF range. The exact reason for this is unknown. However, it could be due to higher estimation of end-diastolic volumes/counts by the planar camera due to incorrect septal separation or overlap in left and right ventricles (31). It has also been suggested that the other possible causes include the differences in acquisition parameters, reorientation software, and image filtering between the cameras (20). 

 The strength of correlation in LVEF values was just moderate (p<0.001) between 2D-echo and the MUGA scans: planar-NaI (r=0.56); CZT-auto (r=0.54); CZT-manual (r=0.56). The Bland–Altman range between 2D-echo and CZT was 35-37%, and between 2D-echo and planar-NaI 33% (-19.7 to +13.6%). The overestimation of ejection fraction by the MUGA scans would be the main reason that there is weak correlation between 2D-echo and nuclear ejection fractions. As seen in the plots in Figure 5 (a, b, and e), the limits of agreement are wide and there is a proportional bias (p<0.05; Table 5). The results exclude any interchangeability of results between 2D-echo and radionuclide scans, which are in line with earlier observations reported in previous studies (9, 16).

***Interobserver variability***

 Our results are in good conformity with the findings of Jensen et al. (20, 33) that the CZT camera has excellent inter-observer reliability and low variability (Table 7). The highest inter-observer reliability was for CZT-auto followed by planar-NaI. The better resolution, faster imaging time, reduced radiopharmaceutical dose, and the established high interobserver reliability as concurred by this study, show that CZT can replace planar to assess LVEF (31).

***RVEF range among breast cancer patients***

 Echocardiography can be used to measure RVEF, although first-pass radionuclide ventriculography is the preferred method. The MUGA scan using planar NaI camera in the left anterior oblique projection (LAO) under-estimates the RVEF due to overlap with right atrium and right ventricle and this method is not recommended by the European Association of Nuclear Medicine and European Society of Cardiology (EANM/ESC) guidelines for radio-nuclide imaging of cardiac function, (2008). 

 According to these guidelines, the mean RVEF at rest for a normal population is 52%, and RVEF dysfunction is defined as RVEF <50%. The new cardiac dedicated CZT camera is capable of measuring RVEF more accurately than the planar camera. The mean RVEF value among breast cancer patients in this study assessed by CZT-auto was 41.5±8.6%, with a normal reported range of 19-63% and these values are in agreement with those reported in the literature, viz: 49.5±11.0% (31); 42.2±8.0% (range 26.8-62.0) (33), 49.5%±10.1%(34).

***Overestimation of LVEF in small heart volumes***

 It has been noted that LVEF is often overestimated in women with small LV cavity size (35), and specifically when ESV<20 mL (36-39). A metanalysis (40) comparing ECG-gated SPECT and cardiac MRI with 164 subjects from 9 studies involving various populations (Japan, USA, Germany and Netherlands) also used similar cutoff values. Kakhki et al. (41), showed that LV function parameters using gated SPECT can be affected by patient populations, different acquisition parameters and processing methods. 

 This Iranian study found that 85.4% of their subjects had ESV<25ml, of which 91% of them were women. Since the vast majority of small heart volume definitions in literature are based on gated SPECT data, population and gender based definitions for small LV cavity size using MUGA scans with both planar and CZT cameras are needed, as a study by Khalil et al. (42) showed that gated SPECT methods have moderate to poor correlations in addition to wide agreement limits with gated blood pool studies in patients with small hearts.

 Left ventricular volume measurement is known to be affected by reconstruction parameters, the reconstruction algorithm, filtering, and zooming, besides absolute ventricular size (43-45). The cause of the overestimation of LVEF in small heart volumes has been examined by several studies (35,46) using myocardial perfusion scans and planar MUGA scans, in which it has been noted that the software edge detection algorithms can often lead to variations in the calculated LVEF. Despite the higher spatial, contrast, and energy resolution, and the true volumetric definition offered by the 3D CZT camera, the overestimation of volume measurements and LVEF, could be due to the partial volume effect (46) seen with these cameras. Applying smoothing reconstruction filters was also shown to lower left ventricular volumes and result in higher ejection fraction values (46).

**Figure 6:**
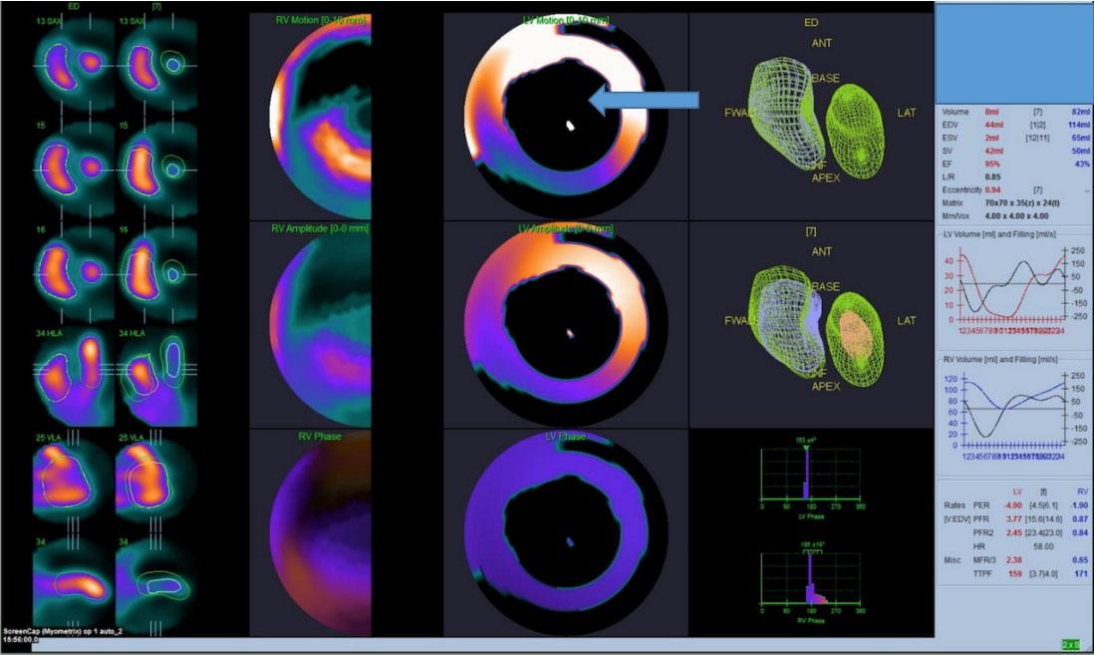
CZT automatic processing results in QBPS software of a patient with ESV value <20mL (2mL in this image) showing overestimated LVEF value of 95% and with regional wall motion detection error (**blue arrow**). The patient had no cardiac rhythm abnormality, and clinically no left chest wall lesion obscuring or causing attenuation artifacts. ECG showed no left ventricular hypertrophy; radiolabelling efficiency was >90%

**Figure 7:**
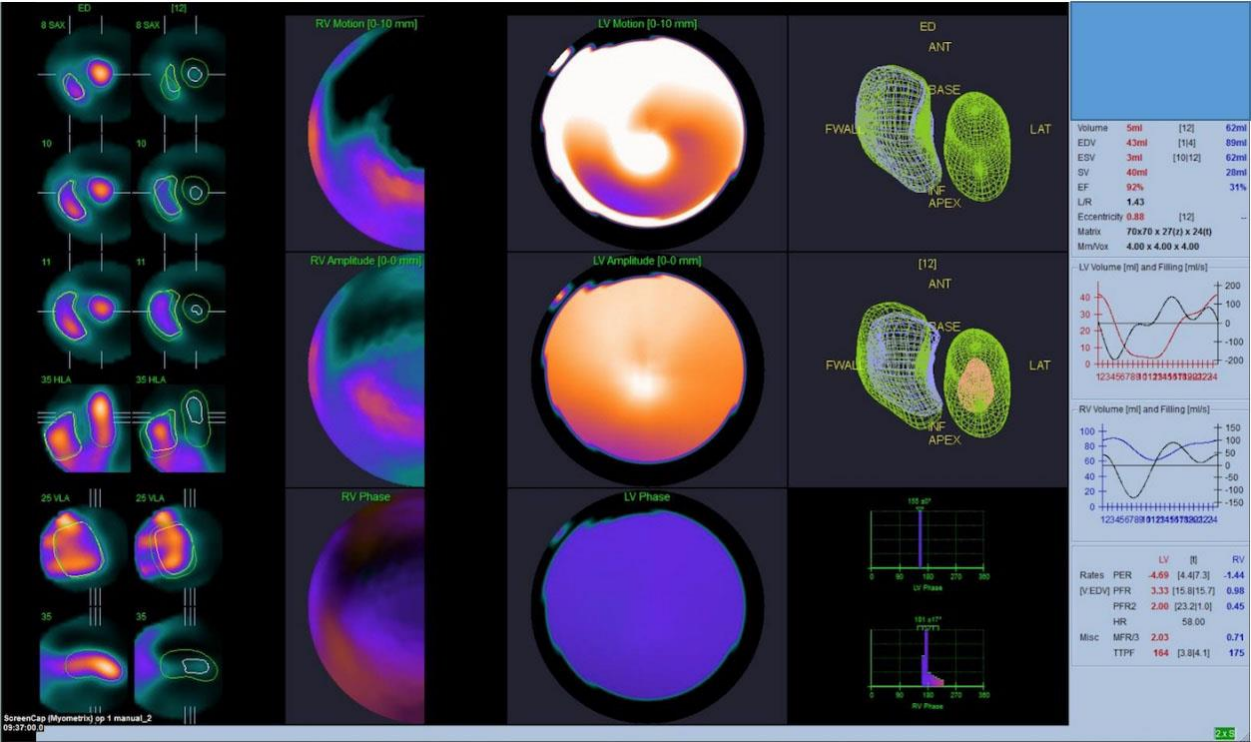
CZT camera QBPS software showing overestimation of LVEF (>80%) in small heart volumes (ESV<20mL) with no regional wall motion error

 A large defect in wall motion analysis was observed in our study for some of the patients with LVEF >90% (indicated with blue arrow in Figure 6), though not for all cases with LVEF exceeding 90% (Figure 7). It can be expected that there would be an exaggerated motion of the myocardium to maintain an adequate stroke volume which could affect the acquisition parameters and QBPS processing software to analyze the LVEF and create wall motion errors. 

 Given that the sample size of patients with low ESV values in this study is small, it is not possible to draw any statistical significance to our results. A further study involving a larger cohort of patients with small ESV volumes is warranted to better understand the limitations of the MUGA scan. However, our findings have important clinical implications for the management of cardiotoxic patients, as there may be a cohort of patients undergoing cardio-toxic drugs with small hearts, and the overestimation of their LVEF by CZT camera or planar NaI camera may give rise to false-negative results with higher than normal LVEF values and show an apparent improvement in LVEF. These patients would then require another modality to assess the LVEF.

***Left breast attenuation***

 The pitfalls of MUGA imaging may include attenuation defects that can be caused by large breasts, breast prosthesis, breast tumour, pericardial or pleural effusion, mediastinal adenopathy or tumours, and mediastinal fat (47). As the MUGA scan measures changes in radioactivity in the left ventricle between end-diastole and end-systole, soft tissue attenuation artifacts can reduce the detected counts and affect the LVEF measurement. Patients with large breasts in particular may show a photopenic U-halo in the left anterior oblique view of the MUGA scan. An anterior image and lateral oblique tilt view may be needed to exclude the breast attenuation artefact (48).

 A good acoustic window is required to perform any echocardiographic method, as it allows adequate visualisation of the blood/endocardial border to allow accurate measurement/tracing. Poor image quality is often observed in obese patients, and also in patients with large breast tumours, and patients with limited space between the ribs. Studies have reported that LVEF could not be determined using the modified Simpson 2D-echocardiography method in 31–38 % of such patients due to poor image quality (35).

 As seen in Table 8, the mean LVEF values measured by 2D-echo and the MUGA scans were comparable (p>0.05) irrespective of the presence or absence of left breast tissue. The objective of this research, however, was not to study the effect of attenuation on the measurement of LVEF in patients that had different types of breast cancer surgery, but it is an area worthy of exploration in a future studies with larger number of patients in each group.

## Limitations

 In a real-world setting, the majority of the breast cancer patients are females, and when planned for potential cardiotoxicity treatment, generally tend to be fit for treatment. This is evidenced by the normal range of LVEF seen in almost all the cases in our study involving only female patients, with very few among them having LVEF <40%. Our findings must therefore be interpreted in the context of the study design, which was a relatively small, prospective, single-center study subject to referral bias. No follow-up studies for the same patients were included. Patients with fungating or large T4 breast lesions were not included in the study as they would have a poor echo window and would not allow for a comparative study to be performed. Another limitation was that no comparative study was made with cardiac MRI which is the current gold standard. 

## Conclusion

 This study showed only a moderate strength of correlation between 2D-echocardiography and MUGA scans using both the planar NaI and CZT cameras. All three modalities also showed suboptimal limits of agreement (>15%) between them. Their results are thus not interchangeable and, therefore, it is recommended that the same chosen modality be used for serial measurements. Ideally, each centre should validate the correlation and agreement in their own institution so as to reduce the need for multiple studies that would add to the cost.

***New Knowledge Gained***

 LVEF measurements using planar and CZT camera are not interchangeable; however, CZT camera offers true volumetric definition of the ventricular volumes due to its 3D acquisition as opposed to count based 2D data offered by the planar camera. Despite the higher count sensitivity and spatial resolution offered by the CZT camera, both cameras showed similar inter-observer reliability.

## List of Abbreviations

 CZT-auto: Cadmium zinc telluride camera operated in automatic mode

CZT-manual: Cadmium zinc telluride camera operated in manual mode

2D-echo: Modified Simpson’s method 2D Echo-cardiography

EDV: End-diastolic volume 

ESV: End-systolic volume

LVEF: Left ventricular ejection fraction

MBq: Megabecquerel

MUGA: Multigated acquisition scan

pNaI: Planar Sodium iodide

SPECT: Single photon emission computed tomography
